# Design and Development of an Autonomous Underwater Helicopter for Ecological Observation of Coral Reefs

**DOI:** 10.3390/s22051770

**Published:** 2022-02-24

**Authors:** Jing Zhou, Nanxi Zhou, Yuchao Che, Jian Gao, Liming Zhao, Haocai Huang, Ying Chen

**Affiliations:** 1 Hainan Institute, Zhejiang University, Sanya 570203, China; jingzhou@zju.edu.cn (J.Z.); hchuang@zju.edu.cn (H.H.); 2 Polytechnic Institute, Zhejiang University, Hangzhou 310015, China; 22160113@zju.edu.cn (N.Z.); gjsy1202@163.com (J.G.); 3 College of Electrical Engineering, Zhejiang University, Hangzhou 310027, China; cyc521521@zju.edu.cn (Y.C.); 0921201@zju.edu.cn (L.Z.); 4 Ocean College, Zhejiang University, Zhoushan 316021, China

**Keywords:** autonomous underwater vehicle, disk-shaped, sensors, dynamic performance, Fuzzy-PID

## Abstract

Real-time status monitoring is an important prerequisite for coral reef ecological protection. Existing equipment does not provide an ocean observation platform with adequate mobility and efficiency. This paper describes the design considerations of a proposed autonomous underwater helicopter (AUH) dedicated for ecological observation of coral reefs, including the system architecture, electronic devices, sensors and actuators, and explains the path control algorithm and controller to follow a specific path for ocean exploration. The structure and dynamic model of the AUH are first introduced, and then the corresponding simplification is made for motion analysis. Furthermore, computational fluid dynamics (CFD) simulation is carried out to evaluate the dynamic performance of the AUH. Fuzzy-PID control algorithm is utilized to achieve a good antidisturbance effect. In order to validate the performance of the proposed underwater vehicle, a field test was performed, and results confirmed the feasibility of the proposed prototype.

## 1. Introduction

Coral reefs, known as “underwater rainforests”, are the most biodiverse ecosystems in the world and provide considerable economic, ecological and social benefits for human beings. With the increasing intensity of social and marine economic activities in coastal areas, coral reefs are facing the threat of severe damage [1]. Real-time observation is an important prerequisite for the rescue and protection of coral reefs; it helps to understand the status of coral reef systems, identify relative impact factors and evaluate the effectiveness of conservation measures, so as to put forward suggestions for further improvement on protection strategies. Traditional coral observations are either carried out by divers carrying cameras and samplers at the coral reef site or by fixed observation platforms with scientific sensors [2,3], which have significant limitations in terms of observation scale and efficiency. With the development of ocean technology, underwater vehicles equipped with professional observation equipment are coming into application in the ocean observation field [4,5,6,7,8,9,10,11]. However, existing underwater vehicles do not provide an ideal platform for large-scale and long-term observation of coral reefs. More specifically, (i) while an autonomous underwater vehicle (AUV) mainly works in the upper water column, the observation of coral reefs requires the underwater vehicle to be able to reach the seafloor and hover at a fixed point; (ii) operation in the complex water flow near the seabed puts forward higher requirements for the maneuverability of the underwater vehicle.

In view of the problems mentioned above, a seafloor-resident mobile observation platform, Coral-AUH, is designed and developed in this paper. As a disk-shaped underwater vehicle, the AUH has experienced several generations of iteration and come into application in various fields [12,13,14,15,16,17,18]. Coral-AUH inherits the super maneuverability and seafloor-resident capability; meanwhile, it carries a camera and multiple sensors to detect environmental data such as pH, conductivity, dissolved oxygen, turbidity and chlorophyll in real time, which helps scientists to determine the health status of corals more accurately. The advent of Coral-AUH will provide a large-scale and long-term observation platform for coral reef ecological observation.

The rest of this paper is arranged as follows: The general configuration of Coral-AUH is discussed first, and then a kinematic model and motion analysis are presented. After that, computational fluid analysis evaluating the hydrodynamic performance of Coral-AUH is presented. The control system is then described, and the results of a pool test validating the feasibility of the control algorithm are presented. Finally, the results of a field test verifying the observation capability and reliability of Coral-AUH are presented.

## 2. Configuration of Coral-AUH

### 2.1. System Architecture

**Figure 1:**
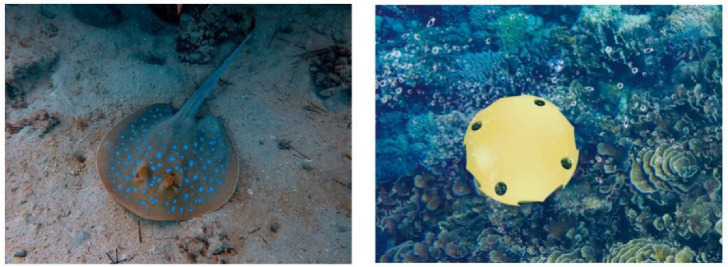
The shape of the AUH inspired by bottom-dwelling stingray.

Inspired by the bottom-dwelling stingray (Figure 1), we designed the disk-shaped autonomous underwater helicopter (AUH), a new member of the AUV family, which is suitable for seafloor-resident observation and operation. Over the decades, a series of AUH prototypes have been developed for various scenarios, and in this paper, Coral-AUH is designed and implemented as the underwater mobile platform for coral observations. Coral-AUH measures 0.5 m × 0.5 m × 0.32 m, weighs 12 kg, is neutrally slightly positive-buoyant, and can swim for 60 min at a time. The designed maximum speed is 2 kn and the maximum diving depth is 200 m.

**Figure 2:**
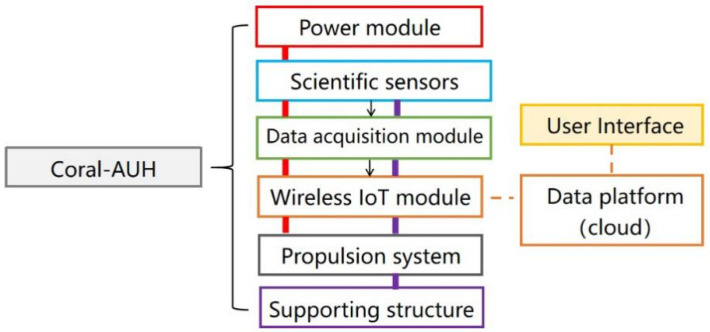
System architecture of Coral-AUH.

The system overview of Coral-AUH is shown in Figure 2. It mainly consists of the shell, propulsion system, power module, data acquisition module, scientific sensor group, wireless Internet of Things module, etc. The power module provides power for functional units on Coral-AUH, while the supporting structure offers a physical connection for all modules. The scientific sensor group is mounted in the frame and includes the intelligent pH (PH) electrode, intelligent conductivity (EC) electrode, intelligent dissolved oxygen (DO) electrode, intelligent turbidity electrodes, self-cleaning chlorophyll digital sensors, etc. (The selection of scientific sensor group was determined according to suggestions from professional coral observation and study team in Nanhai Institute of Chinese Academy of Sciences.) There are two sealed cabins, which isolate inner electronic devices from water. The upper sealed cabin contains a motion control module, data acquisition module and wireless Internet of Things module, and the lower sealed cabin contains power supply components such as batteries. The data acquisition control module is connected with the scientific sensor assembly through the RS485 bus, and the data acquisition control module can read the water quality data fed back by the sensor in real time and perform corresponding processing by sending control signals to the water quality sensor assembly according to the set time sequence.

**Figure 3:**
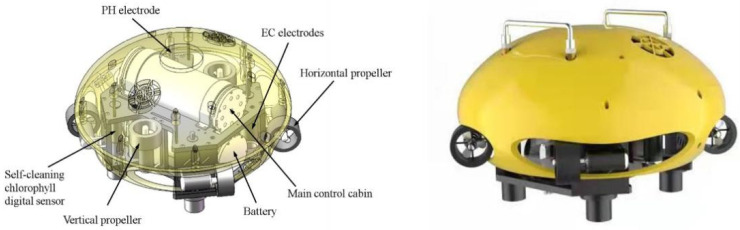
System overview of Coral-AUH.

As shown in Figure 3, the main body of Coral-AUH adopts a frame structure, which is convenient for the installation and fixation of the load devices, more specifically, the scientific sensors. The outer casing is an arc-shaped dome cover, which helps to improve the hydrodynamic performance, and an opening channel for each propeller is arranged on the casing to allow for the water to flow through. The propeller group contains a total of six propellers, including four horizontal propellers, which are symmetrically installed around the mounting bracket to provide torsion force rotating around the vertical central axis and thrust force for horizontal movement. The four propellers are decoupled and can be independently controlled for advance, retreat and yaw. The propulsion direction of the remaining two propellers is the vertical direction. They are symmetrically installed on both sides of the mounting bracket to provide the thrust of the vertical motion of the underwater drone for controlling snorkeling. This propeller layout can improve the space utilization rate and make the designed AUH structure more compact and smaller in appearance. Meanwhile, through staggered arrangement, the flow channels of each propeller do not interfere with each other.

### 2.2. Kinematic Modelling and Motion Analysis

**Figure 4:**
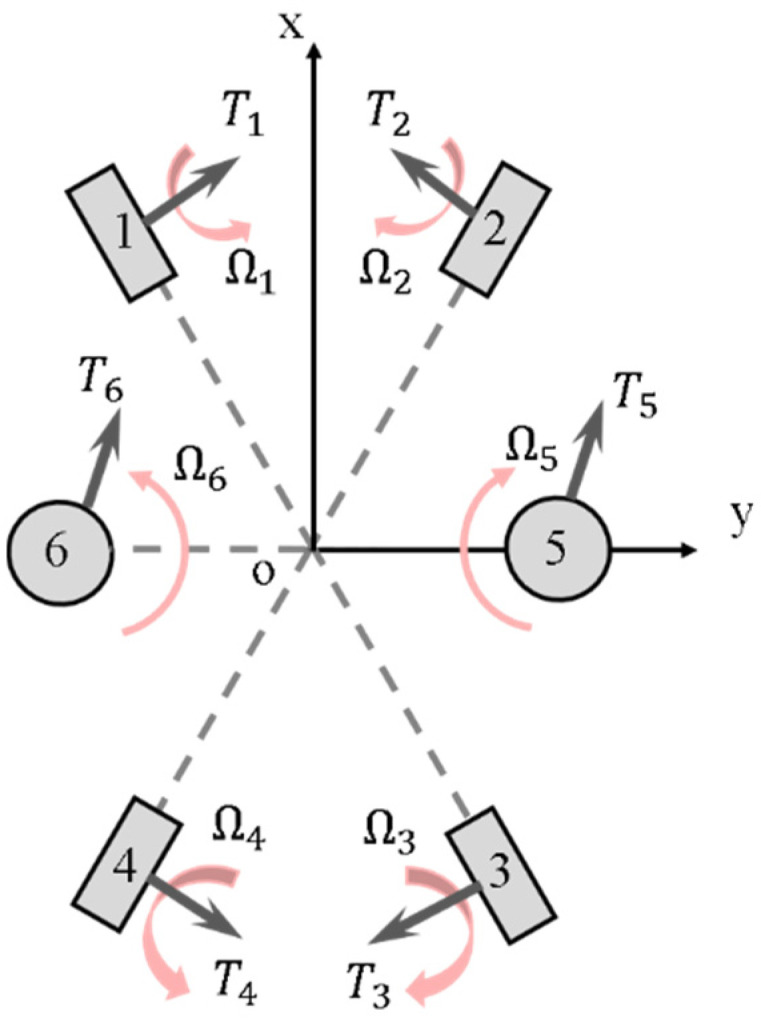
Schematic diagram of the propeller layout.

As the underwater vehicle designed in this paper is dedicated for coral ecological observation, so one key index is to improve the athletic performance, especially its maneuverability, compared with the traditional torpedo-type autonomous underwater vehicle. Considering the requirement for mobility, as well as the special limitations, the layout of propellers was designed as shown in Figure 4. The six propellers are decoupled from each other, and all of them can independently control the trajectory of the AUH by changing the rotational speed.

**Figure 5:**
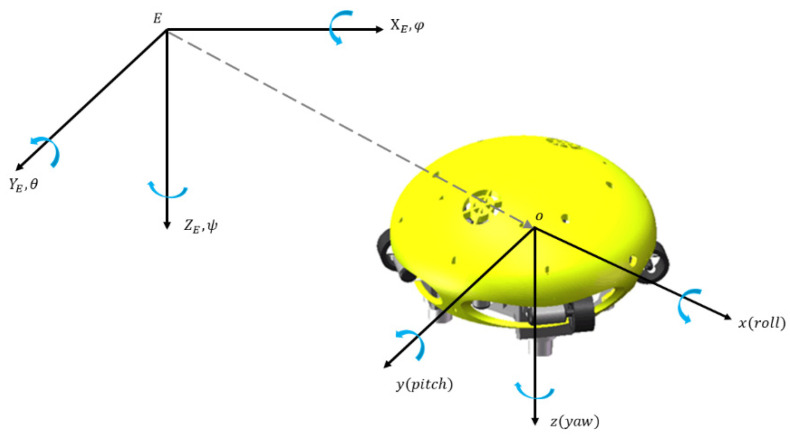
Coordinate frame system.

Before establishing the kinematic model, it is necessary to define the coordinate system. As shown in Figure 5, there are two coordinate frames: one is the ground coordinate frame (E-X_E Y_E Z_E), also known as the static coordinate frame, which is used as the inertial reference system, and the other is the body coordinate frame (O-xyz). In this paper, the center of buoyancy is selected as the origin O of the coordinate frame, with the body-referenced *x*-axis forward, *y*-axis to port (right) and *z*- axis down.

The definitions concerning six degrees of freedom (DOFs) of Coral-AUH are given in the tables below, three in translations and three in rotations. The variables in the inertial frame and body frame are shown in Table 1 and Table 2.

Based on the variables defined in Table 1 and Table 2, the linear velocity and angular velocity of Coral-AUH can be expressed as $V_{E}={\left[\dot{\xi \;}\dot{\eta \;}\dot{\zeta }\right]}^{T},\;\;V_{O}={\left[u\;v\;w\right]}^{T}$, $\;\omega_{E}={\left[\dot{\varphi \;}\dot{\theta \;}\dot{\psi }\right]}^{T}\;$ and $\omega_{O}={\left[\dot{p\;}\dot{q\;}\dot{r}\right]}^{T}$. The linear velocity and angular velocity can be converted through the coordinate transformation matrix [19], as shown in Equations (1) and (2). Equation (3) is the specific expression of the speed conversion matrix *R_V_*

(1) $$\left[\begin{array}{c} \dot{\xi \;}\\ \dot{\eta }\\ \dot{\zeta } \end{array}\right]=R_{V}\left[\begin{array}{c} u\\ v\\ w \end{array}\right]$$

(2) $$\left[\begin{array}{c} \dot{\psi }\\ \dot{\theta }\\ \dot{\varphi } \end{array}\right]=\left[\begin{array}{ccc} 0 & \sec\theta \cos\varphi & -\sec\theta \sin\varphi \\ 0 & \sin\varphi & \cos\varphi \\ 1 & -\tan\theta \cos\varphi & \tan\theta \sin\varphi \end{array}\right]\left[\begin{array}{c} p\\ q\\ r \end{array}\right]$$

(3) $$R_{V}=\left[\begin{array}{ccc} \cos\psi \cos\theta & \cos\psi \sin\theta \sin\phi -\sin\psi \cos\phi & \cos\psi \sin\theta \cos\phi +\sin\psi \sin\phi \\ \sin\psi \cos\theta & \sin\psi \sin\theta \sin\phi +\cos\psi \cos\phi & \sin\psi \sin\theta \cos\phi -\cos\psi \sin\phi \\ -\sin\theta & \cos\theta \sin\phi & \cos\theta \cos\phi \end{array}\right]$$

Theoretically, six propellers can control the motion of the underwater vehicle in six DOFs. However, in practical application scenarios, motion in all DOFs is not often required. As for Coral-AUH, the three degrees of freedom of forward, steering and lifting are sufficient to meet the needs of coral observations. Based on the kinematic model above, the motion analysis of Coral-AUH is carried out in these three DOFs.

**Figure 6:**
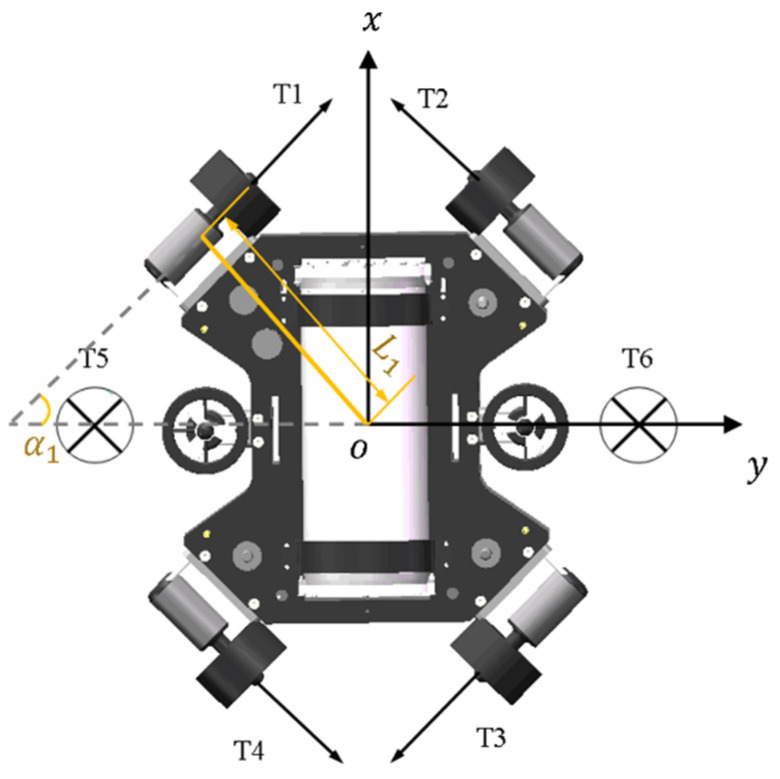
Free body diagram.

The free body diagram of the six propellers is shown in Figure 6.

The expression corresponding to the free body diagram produced by the propellers can thus be obtained:

(4) $$\tau =\left[\begin{array}{c} X\\ Y\\ Z\\ K\\ M\\ N \end{array}\right]=\left[\begin{array}{c} sin\;\alpha_{1}\left(T_{1}+T_{2}-T_{3}-T_{4}\right)\\ cos\;\alpha_{1}\left(T_{1}-T_{2}-T_{3}+T_{4}\right)\\ T_{5}+T_{6}\\ L_{5\;}\left(-T_{5}+T_{6}\right)\\ 0\\ L_{1\;}\left(-T_{1}+T_{2}-T_{3}+T_{4}\right) \end{array}\right]$$

Considering the practical application, this paper only considers three DOFs. The force generated by the propeller can be calculated by $T_{i}$ = $K_{f}\Omega_{i}{}^{2}$, where $K_{f}$ is the propeller coefficient and $\Omega_{i}$ is the angular velocity of the i-th propeller. Equation (4) can be simplified as

(5) $$\tau =\left[\begin{array}{c} X\\ Z\\ N \end{array}\right]=\left[\begin{array}{c} \sin\alpha_{1}\left(T_{1}+T_{2}-T_{3}-T_{4}\right)\\ T_{5}+T_{6}\\ L_{1\;}\left(-T_{1}+T_{2}-T_{3}+T_{4}\right) \end{array}\right]=\left[\begin{array}{c} K_{f}\sin\alpha_{1}\left(\Omega_{1}{}^{2}+\Omega_{2}{}^{2}-\Omega_{3}{}^{2}-\Omega_{4}{}^{2}\right)\\ \;\;\;K_{f}\left(\Omega_{5}{}^{2}+\Omega_{6}{}^{2}\right)\\ K_{f}L_{1\;}\left(\Omega_{2}{}^{2}+\Omega_{4}{}^{2}-\Omega_{1}{}^{2}-\Omega_{3}{}^{2}\right) \end{array}\right]$$

In this paper, the Newton–Euler equation [20] is used to establish the dynamic model. On the horizontal plane, the AUH kinetic model can be expressed as

(6) $$J\dot{\omega }+\omega *\left(J\omega \right)+C_{gyro}+M_{f}+K_{\zeta }+M_{C}=\tau$$

where J is the total inertial of the vehicle, ω*(Jω) is the centripetal force and $C_{gyro}\;$ is the gyroscopic effect moment generated by the propeller due to the rotation of the propeller. $M_{f}$ is water resistance or moment, which can be calculated by $M_{f}=\frac{1}{2}\rho C_{f}A_{f}V^{2}$, where ρ is the fluid density, $C_{f\;}$ is the water resistance coefficient, $A_{f}$ is the wet surface area of the AUH and V is the flow rate. $K_{\zeta \;}$ is the force and moment produced by the gravity and the buoyancy. $M_{C}$ is the force and moment generated by the optical cable to the AUH, which can be written as $M_{C}=M_{p}+M_{f}=\frac{1}{2}\rho C_{c}A_{C}V_{f}{}^{2}$, where $C_{c}$ is the resistance coefficient, $A_{C}$ is the wet area of the cable and $V_{f}$ is the water velocity. τ is the force and moment generated by the propeller.

Since the gyroscopic effect has little influence on underwater objects, it is not considered in this paper, and the resistance of the optical cable to the AUH is ignored. Combined with the snorkeling motion in the vertical direction, the equation of motion under three degrees of freedom can finally be simplified as

(7) $$m\ddot{\xi }=K_{f}\sin\alpha_{1}\cos\psi \left(\Omega_{1}{}^{2}+\Omega_{2}{}^{2}-\Omega_{3}{}^{2}-\Omega_{4}{}^{2}\right)-\frac{1}{2}\rho C_{fH}A_{H}{\dot{\xi }}^{2}\cos\psi$$

(8) $$m\ddot{\zeta }=-K_{f}\left(\Omega_{5}{}^{2}+\Omega_{6}{}^{2}\right)-\frac{1}{2}\rho C_{fV}A_{V}{\dot{\zeta }}^{2}+K_{\zeta }$$

(9) $$J_{Z}\ddot{\psi }=K_{f}L_{1\;}\left(\Omega_{2}{}^{2}+\Omega_{4}{}^{2}-\Omega_{1}{}^{2}-\Omega_{3}{}^{2}\right)-C_{R}R\dot{\psi }\;$$

where $\;C_{fH}$ and $C_{fV}$ are the drag coefficients in the horizontal and vertical directions obtained according to the hydrodynamic analysis, which will be introduced in detail in Section 2.3. $A_{H}$ and $A_{V}$ respectively represent the wet area in the horizontal and vertical directions of the AUH. $C_{R}$ is the general water resistance coefficient when the AUH rotates. R is the turning radius of the designed AUH. The input control variable of the Coral-AUH is the angular velocity of the i-th propeller. This section clarifies the dynamic law and its quantitative expression for Coral-AUH, which prepares for the subsequent parameter setting of the control system.

### 2.3. CFD Simulation

In motion analysis, the water resistance is an important variable affecting the kinematic performance of Coral-AUH, which requires accurate calculation. However, due to the complex underwater environment, the water resistance of Coral-AUH when working underwater is not linear and can hardly be obtained directly. CFD software proves to be an effective tool to evaluate the resistance of underwater vehicles, and the simulation results can be used to optimize the external dimension parameters of the Coral-AUH afterward. The diversion shell of the AUH is mainly utilized to protect the internal equipment of the helicopter from the water flow in the marine environment and the impact damage of various debris; meanwhile, its hydrodynamic profile helps to reduce the fluid resistance in the operation process so as to reduce the power consumption.

In this paper, ANSYS-FLUENT is utilized as the CFD simulation tool. To ensure calculation accuracy and save computing resources, the RNG k-**ε** model is used to establish an outer drainage basin of 4 m × 4 m × 10 m. The velocity inlet and pressure outlet boundary conditions are established, and the rest are set as no-slip walls. Considering the complexity of the model, unstructured grid is adopted globally. The boundary layer is refined near the wall of the inner watershed, and the minimum grid size is 2 mm. The RNG model is one of the widely used two-equation turbulence models; it solves two transport equations and simulates Reynolds stress by the eddy viscosity method.

(10) $$\frac{\partial \rho }{\partial t}+\frac{\partial }{\partial x_{i}}\left(\rho u_{i}\right)=0$$

(11) $$\frac{\partial }{\partial t}\left(\rho u_{i}\right)+\frac{\partial }{\partial x_{j}}\left(\rho u_{i}u_{j}\right)=\frac{\partial p}{\partial x_{i}}+\frac{\partial }{\partial x_{j}}\left[\mu \left(\frac{\partial u_{i}}{\partial x_{j}}+\frac{\partial u_{j}}{\partial x_{i}}-\frac{2}{3}\delta_{ij}\frac{\partial u_{l}}{\partial x_{l}}\right)\right]+\frac{\partial }{\partial x_{j}}\left(-\rho \bar{u_{i}^{’}u_{j}^{’}}\right)$$

The RNG *k*-*ε* model [21] is based on model transport equations for the turbulence kinetic energy (k) and its dissipation rate (*ε*), which are obtained from the following transport equations:

(12) $$\frac{\partial }{\partial t}\left(\rho k\right)+\frac{\partial }{\partial x_{i}}\left(\rho ku_{i}\right)=\frac{\partial }{\partial x_{j}}\left(\alpha_{k}\mu_{eff}\frac{\partial k}{\partial x_{j}}\right)+G_{k}+G_{b}-\rho \varepsilon -Y_{M}+S_{k}$$

(13) $$\frac{\partial }{\partial t}\left(\rho \varepsilon \right)+\frac{\partial }{\partial x_{i}}\left(\rho \varepsilon u_{i}\right)=\frac{\partial }{\partial x_{j}}\left(\alpha_{\varepsilon }\mu_{eff}\frac{\partial \varepsilon }{\partial x_{j}}\right)+C_{1\varepsilon }\frac{\varepsilon }{k}\left(G_{k}+C_{3\varepsilon }G_{b}\right)-C_{2\varepsilon }\rho \frac{\varepsilon^{2}}{k}-R_{\varepsilon }+S_{\varepsilon }$$

In these equations, $G_{k}$ represents the generation of turbulence kinetic energy due to the mean velocity gradients and $G_{b}$ is the generation of turbulence kinetic energy due to buoyancy. $Y_{M}$ represents the contribution of the fluctuating dilatation in compressible turbulence to the overall dissipation rate. $C_{1\varepsilon },\;C_{2\varepsilon }$ and $C_{3\varepsilon }$ are constants and $\sigma_{k}$ and $\sigma_{\varepsilon }$ are the turbulent Prandtl numbers for k and ε, respectively. The quantities $\alpha_{k}$ and $\alpha_{\varepsilon }$ are the inverse effective Prandtl numbers for k and ε, respectively. $S_{k}$ and $S_{\varepsilon }$ are user-defined source terms.

The numerical solver uses the pressure-based Semi-Implicit Method for Pressure Linked Equations (SIMPLE), which iteratively decouples the calculation of velocity and pressure. The condition for iterative calculation to stop in each case is that the residuals of each parameter are less than $10^{-3}$.

**Figure 7:**
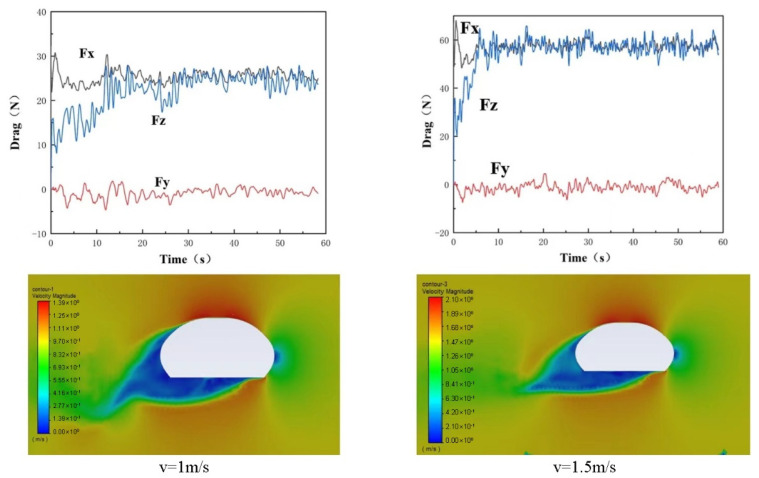
The resistance diagram and the velocity contour.

Two typical translation and rotation velocities were simulated, and the resistance obtain could be input into the motion control analysis. The resistance diagrams and the velocity contour of the Coral-AUH are shown in Figure 7.

Comparing the resistance of the Coral-AUH in all directions under the conditions of the incoming flow speeds of 1 and 1.5 m/s, the resistance Fx is about 25 N at 1 m/s and about 55 N at 1.5 m/s. The resistance Fy basically fluctuates up and down around 0 N, and the Coral-AUH receives roughly the same resistance in the *x*-axis and *z*-axis, which may cause rising during the horizontal advance. It can be fine-tuned by the vertical propellers. From the velocity contour, at the speeds of 1 and 1.5 m/s, the surrounding fluid flow is similar and the streamline is good. The flow velocity of the upper fluid and the opposite flow is relatively large and then gradually decreases to both sides of the hull, and the flow velocity of the fluid behind the helicopter is relatively small.

**Figure 8:**
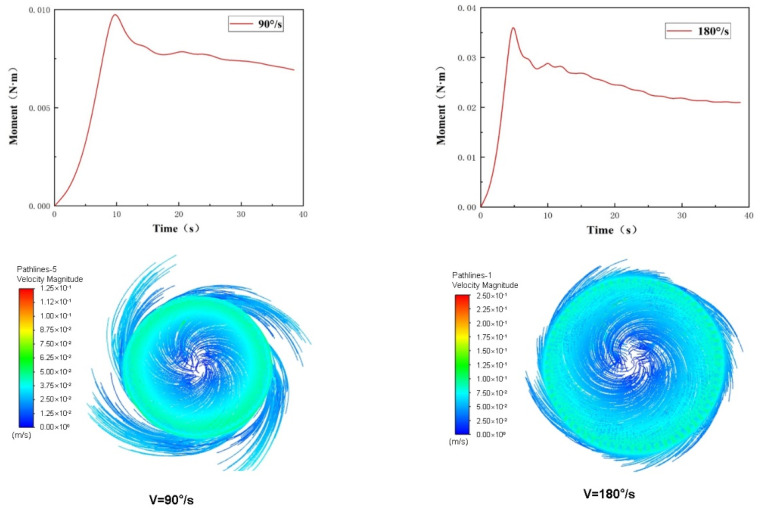
The torque curves and velocity contours.

In addition to translational movement, steering is also a frequent motion in underwater operations. Therefore, the torque of the Coral-AUH when it rotates at different angular speeds perpendicular to *z*-axis was analyzed. The torque variations and velocity contours when angular speed is at 90 and 180°/s are shown in Figure 8.

By comparison, it can be found that the torque is about 0.006 N m at 90°/s and 0.02 N m at 180°/s. It is much smaller than the resistance encountered when moving horizontally. It can better meet the requirements of flexible steering and maneuverability of underwater helicopters during movement. Meanwhile, the resistance in the horizontal direction of the underwater helicopter is small and the action is flexible, while the resistance in the vertical direction is large, which can also ensure the stability of its navigation process.

## 3. Guidance and Control Method

### 3.1. Guidance and Actuators

**Figure 9:**
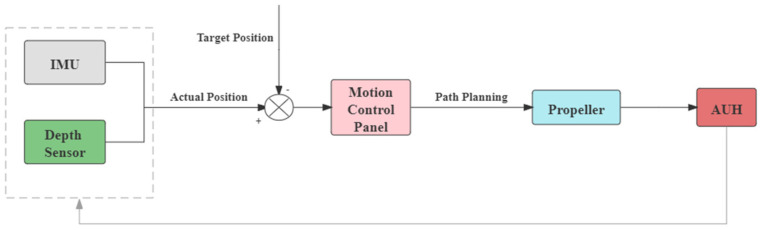
Flow chart of the guidance and actuation system.

The positioning system of the Coral-AUH consists of an inertial measurement unit (IMU) and depth sensor (Figure 9). An AUH can be equipped with an ultrashort baseline (USBL) system for underwater acoustic positioning. The USBL system is usually expensive and heavy, and acoustic positioning is not the focus of this paper. Relative displacement and angular variation data are read from IMU to locate the AUH and to verify the feasibility of motion actuators.

The motion actuators in Coral-AUH are the horizontal and vertical propellers. The motion control panel perceives data from sensors and outputs motion commands after position calculation. The inertial measurement unit (IMU) measures the three-axis attitude angle and acceleration rate of the Coral-AUH. The depth sensor measures the depth of the AUH. Having received data from the positioning system, the motion control panel then calculates the actual position of the Coral-AUH and compares it with the target position and sends commands to the attitude adjustment system and propulsion system accordingly.

### 3.2. Motion Control

The motion of the AUH requires the collaborative work of six brushless DC (BLDC) motors. An STM32 generates PWM at a certain frequency and duty cycle, which is utilized as drive signals for the propeller group. The control command comes from the data collected from sensors and remote control signals. The IMU module is composed of an ITG3205 triaxial gyroscope, an ADXL345 triaxial accelerometer and an HMX5883L triaxial magnetic field sensor. PID closed-loop control is utilized to ensure that the Coral-AUH successfully completes the desired motion.

**Figure 10:**
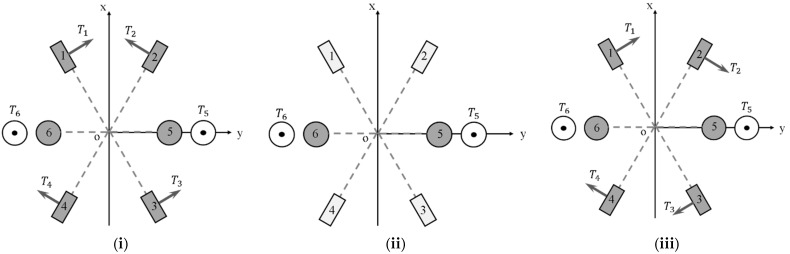
The operating modes of the propellers. (**i**) forward and backward; (**ii**) up and down; (**iii**) left and right steering.

In the practical application process of the AUH, three modes are mainly involved: forward and backward, up and down, and steering. The operating modes of the propellers will be introduced below (Figure 10).

(i) Forward and backward

When moving forward, the four horizontal propellers maintain the same rotation speed. Propellers 1 and 4 generate a clockwise force perpendicular to the central axis, and propellers 2 and 3 generate a counterclockwise force perpendicular to the central axis. At this time, the force of the four horizontal propellers in the *y*-axis direction is just offset. The resultant force is the forward thrust. The principle of moving backward is omitted since it is similar to that of moving forward.

(ii) Up and down

When the two vertical propellers simultaneously generate the same upward or downward force according to the speed requirement, Coral-AUH moves in the vertical direction.

(iii) Left and right steering

When the horizontal propellers in relative position propel in opposite directions, Coral-AUH can realize 360° turning with zero radius.

### 3.3. Fuzzy-PID Control Algorithm

**Figure 11:**
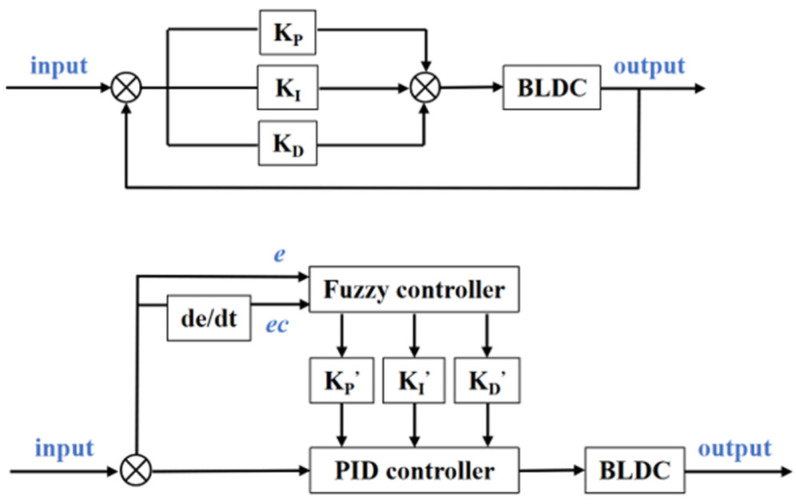
Logic diagrams of traditional PID control and fuzzy-PID control.

The traditional PID controller features high reliability, simple structure and easy parameter adjustment, but it also has some shortcomings; for example, the control accuracy is not sufficient and the anti-interference ability is poor. The parameters in traditional PID control are preset, and when the system is under disturbance, preset parameters cannot be changed, so the performance of the traditional controller cannot meet certain precision. In fuzzy algorithms, parameters can adapt to changes according to certain rules, so a fuzzy-PID control algorithm has better antidisturbance performance than a traditional PID control algorithm. The comparison of fuzzy-PID control and traditional PID control logic diagrams is shown in Figure 11.

The traditional PID control system is mainly composed of a PID controller and a controlled object. In this paper, the control object is a brushless DC motor. According to the given value r(t) of the system and the actual output feedback *c(t)* of the control system, the deviation value of the system e(t)=r(t)−c(t).

(14) $$u(t)=K_{p}\left[e(t)+\frac{1}{T_{i}}\int_{0}^{t}e(t)dt+T_{d}\frac{de(t)}{d(t)}\right]$$

(15) $$u\left(t\right)=K_{p}*e\left(t\right)+K_{I}\sum e\left(t\right)+K_{D}\;{\left[e\left(t\right)-e\left(t-1\right)\right]}_{k}$$

(16) $$\mathrm{In}\;\mathrm{which},\;K_{I}=\frac{K_{p}}{T_{i}};K_{D}=\frac{K_{p}T_{d}}{T}.$$

Fuzzy control is a nonlinear control strategy based on fuzzy reasoning. Its basic principle is to integrate human operation experience and common sense reasoning rules through fuzzy language. When the stability of the system is destroyed, the fuzzy control algorithm can make a judgment according to the current state to maintain stability, so the fuzzy algorithm is integrated into the traditional PID algorithm to form a fuzzy PID controller, which usually achieves a good control effect. As shown in Figure 11, error e and error variation rate ec are input into the fuzzy controller, and KP’, KI’ and KD’ are obtained after calculation. These three environment-variant parameters are input into the PID controller in real time, which facilitates the application under different circumstances.

**Figure 12:**
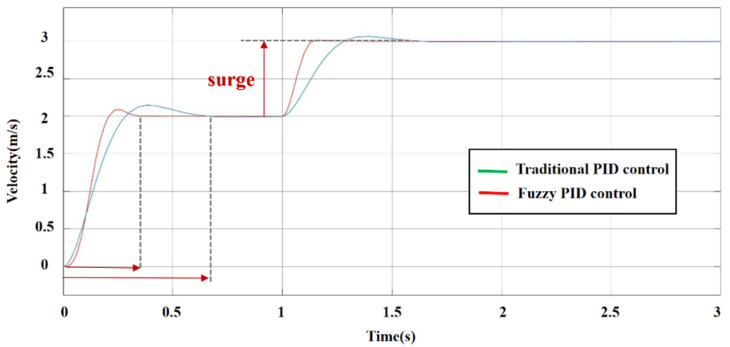
Response curves of traditional and fuzzy PID controllers.

The simulation model was established in MATLAB Simulink. In the traditional PID control model, according to the optimization result, KP is set as 8, KI is set as 0.3 and KD is set as 0.8. In the fuzzy PID control model, fuzzification factors Ke = 2 and Kec = 0.1; defuzzification factors K1 = 0.8, K2 = 120 and K3 = 0.5; and initial PID parameters Kp = 0.6, Ki = 0.8 and Ki = 0.05. The comparison of response curves in traditional PID control and fuzzy PID control is shown in Figure 12. As shown in Figure 10, compared with the traditional PID controller, the fuzzy PID controller has a faster response speed, shorter time to reach the steady state and much less overshoot. When the reference input surges, the response speed of fuzzy PID control is faster in the adjustment process, and there is almost no overshoot, so the controller has a better anti-interference ability.

## 4. Field Test

### 4.1. Motion Test

We performed quantitative tests in a pool to measure the propulsion and attitude adjustment capabilities of the Coral-AUH. The average swimming speed in a straight path was 1.54 m/s (±0.06 m/s), equivalent to 1.5 body lengths per second. The average angular spin rate was 3.14 rad/s (±0.6 rad/s) with a zero turning radius. The average dive speed in the horizontal attitude was 0.72 m/s (±0.07 m/s), equivalent to 0.7 body lengths per second. A sample AUH trajectory is shown in Figure 11, illustrating the ultra-agile swimming motion following preset commands. The AUH can change attitude, direction and depth while exploring the coral reefs, with an average swimming speed of 1.28 m/s (±0.26 m/s) at depths of 0 to 200 m.

**Figure 13:**
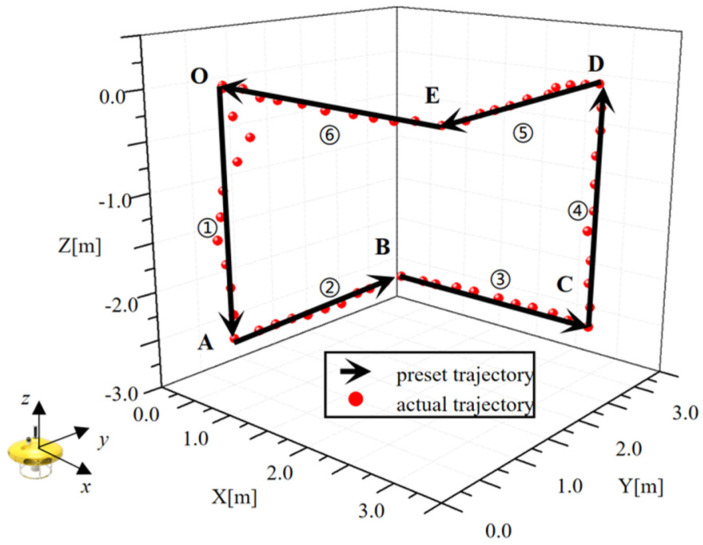
The 3D trajectory of Coral-AUH.

**Figure 14:**
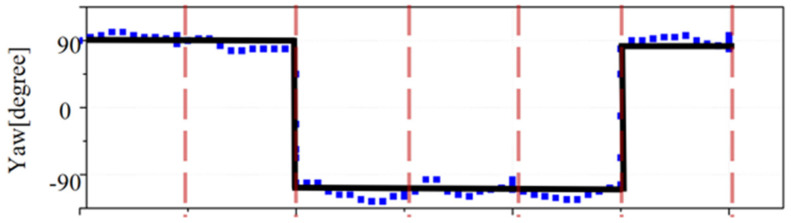
Heading angle of Coral-AUH (yaw).

In order to verify the feasibility of the control algorithm, an experiment to test mobility was carried out in a swimming pool. A preset route was designed and input into the control system of the AUH, and the route was quite challenging as it had sharp turning and attitude adjustment. In the preset task, the AUH is supposed to follow the route through attitude adjustment and vectoring propulsion. As shown in Figure 13, the trajectory started at point O; AUH was designed to dive to Point A and then cruise to point B. After that, AUH turned 90° clockwise and cruised to point C. At point C, AUH came up to point D, and then it cruised to point E. At point E, AUH turned 90° clockwise and finally returned to point O. The whole route mainly involves variations in yaw. A comparison was made between the preset route and the actual route, and the result is shown in Figure 13. Yaw angle was also recorded in real time and compared with the preset value, as shown in Figure 14. The actual route and heading angle agree well with preset values, validating the fuzzy control algorithm.

### 4.2. Field Test

**Figure 15:**
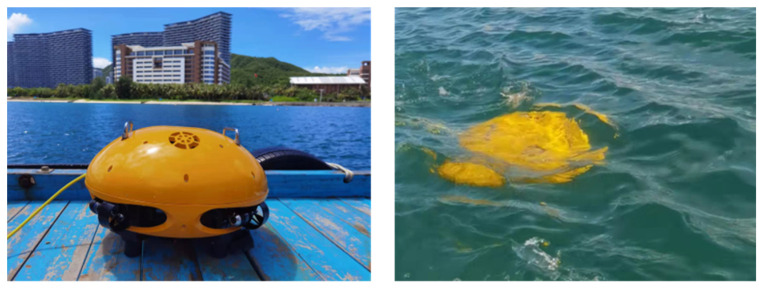
Coral-AUH in field test.

**Figure 16:**
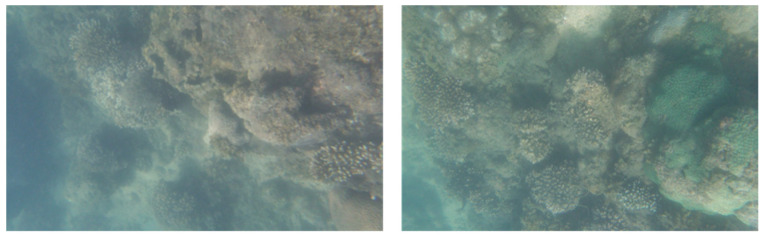
National Coral Conservation Area in Luhuitou.

**Figure 17:**
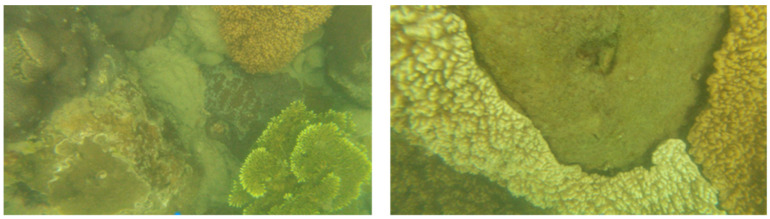
Picture taken under the sea of Dongluo Island.

The function and reliability of Coral-AUH were tested in a field test in the waters of Luhuitou and Yazhou Bay in Sanya City. Coral reef ecological restoration areas located in the Nanhaisuo National Coral Conservation Area and the Dongluo Island Coral Restoration Area were selected as test sites (shown in Figure 15). In the field test, Coral-AUH successfully cruised to the desired site with a preset route. The photos taken by Coral-AUH are shown in Figure 16 and Figure 17, and the water quality data collected by the sensor group are shown in Table 3 and Table 4.

The field test lasted 2 h each time. The maximum depth reached was 30 m. It can be seen from the test results that the Coral-AUH successfully cruised to the coral reef site and hovered there to take photographs; meanwhile, it could detect environmental data such as pH, conductivity, dissolved oxygen, turbidity and chlorophyll in real time. The test results show that the system can improve the accuracy and timeliness of coral monitoring and can meet the long-term stereoscopic observation requirements for coral reefs in a complex seabed ecological environment.

## 5. Conclusions

This paper contributes to the field of undersea robots that can serve as an observatory platform for coral reefs. First, we present the design considerations of a stingray-shaped underwater vehicle that can complete scientific surveys continuously within certain sea areas. Motion analysis and CFD simulation show Coral-AUH has low rotation resistance and superior maneuverability. A fuzzy algorithm is integrated into the traditional PID algorithm to form a fuzzy PID controller, which usually achieves a good antidisturbance effect. Simulation and experimental results show that AUH follows the preset route well and demonstrates excellent maneuverability. Finally, the function and reliability of Coral-AUH were tested in the waters of Luhuitou and Yazhou Bay at the depths of 10 and 30 m, respectively, and the duration of each test lasted for around 2 h. The results show that Coral-AUH can quickly, accurately and efficiently conduct coral observation tasks.

## Figures and Tables

**Table 1:** Parameters in the inertial frame.

Parameter	Displacement	Position Angle
$X_{E}$	ξ	φ
$Y_{E}$	η	θ
$Z_{E}$	ζ	ψ

**Table 2:** Parameters in the body frame.

Vector	Speed (V)	Angular Velocity (Ω)	Force (F)	Torque (M)
x	u	p	X	K
y	v	q	Y	M
z	w	r	Z	N

**Table 3:** Water quality data of National Coral Conservation Area.

Position		pH	Electric Conductivity (uS/cm)	Dissolved Oxygen Concentration (mg/L)	Turbidity (FTU)	Chlorophyll (ug/L)
Eastern Longitude	Northern Latitude					
18°12′42′’	109°28′21”	8.08	47.8	6.32	21.05	3.02
18°12′45′’	109°28′25′’	8.13	46.35	6.17	19.83	3
18°12′46′’	109°28′34”	8.11	47.48	5.93	23.42	2.72
18°12′47”	109°28′38”	8.13	52.64	5.89	16.8	3.39

**Table 4:** Water quality data of Dongluo Island.

Position		pH	Electric Conductivity (uS/cm)	Dissolved Oxygen Concentration (mg/L)	Turbidity (FTU)	Chlorophyll (ug/L)
Eastern Longitude	Northern Latitude					
18°19′32′’	108°59′30′’	8.06	47.8	5.99	20.21	3.2
18°19′28′’	108°59′20′’	8.15	46.35	6.03	35.02	4.81
18°19′42′’	108°59′18′’	8.2	47.48	6.01	15.98	2.91
18°19′44′’	108°59′19′’	8.25	52.64	5.91	16.13	3.23

## Data Availability

All the data supporting the reported results has been included in this paper.
